# A Review on Current Status of Biochar Uses in Agriculture

**DOI:** 10.3390/molecules26185584

**Published:** 2021-09-14

**Authors:** Tara Allohverdi, Amar Kumar Mohanty, Poritosh Roy, Manjusri Misra

**Affiliations:** 1 Bioproducts Discovery and Development Centre, Department of Plant Agriculture, Crop Science Building, University of Guelph, Guelph, ON N1G 2W1, Canada; tallohve@uoguelph.ca (T.A.); poritosh@uoguelph.ca (P.R.); 2 School of Engineering, Thornbrough Building, University of Guelph, Guelph, ON N1G 2W1, Canada

**Keywords:** biochar, soil amendment, agriculture, sustainability

## Abstract

In a time when climate change increases desertification and drought globally, novel and effective solutions are required in order to continue food production for the world’s increasing population. Synthetic fertilizers have been long used to improve the productivity of agricultural soils, part of which leaches into the environment and emits greenhouse gasses (GHG). Some fundamental challenges within agricultural practices include the improvement of water retention and microbiota in soils, as well as boosting the efficiency of fertilizers. Biochar is a nutrient rich material produced from biomass, gaining attention for soil amendment purposes, improving crop yields as well as for carbon sequestration. This study summarizes the potential benefits of biochar applications, placing emphasis on its application in the agricultural sector. It seems biochar used for soil amendment improves nutrient density of soils, water holding capacity, reduces fertilizer requirements, enhances soil microbiota, and increases crop yields. Additionally, biochar usage has many environmental benefits, economic benefits, and a potential role to play in carbon credit systems. Biochar (also known as biocarbon) may hold the answer to these fundamental requirements.

## 1. Introduction

Anthropogenic effects of climate change and unsustainable agriculture have caused drought, fertilizer leaching and lack of food security worldwide [1]. With immediate and looming future problems, biochar may be the key to developing a sustainable future while adding valued products to the circular economy model. Researchers desire a potential solution to improve soil quality by applying biochar for soil amendment and improve the sustainability of agriculture [2]. Aspects of soil that determine good quality include texture, capacity to retain and sustain microbial activity and ability to retain nutrients and moisture [3]. Usually, biochar is used without any treatment, but recent research shows that physically or chemically modified biochar could be applied to improve performance [4]. Biochar has been found to have a positive effect on soil health and plant yields, while keeping the health of soil intact [5]. The ability to increase yields without synthetic fertilizers or soil additives is a challenge for modern sustainable agricultural methods [6]. In order to achieve sustainability, retention of water and nutrients in agricultural soils are fundamental qualities to address [6]. Carbon dioxide (CO_2_) emissions from fossil fuel use is now known to be the major driving force behind climate change [7]. Capturing this atmospheric carbon can attenuate the rising greenhouse gas (GHG) emissions. The agricultural sector is one area in which huge amounts of biochar can be used to store carbon [7]. The way in which soils can act as a carbon sink depends on a few factors (varying porosity namely) but ultimately are able to sequester varying degrees of carbon in soils [4]. In general, most new agricultural land comes from the tropical rainforests that are already under threat of deforestation [8]. Currently, China and the United States are the forerunners in using biochar for agricultural purposes [9]. Even though studies exist on biochar amendment for purposes of agriculture, they generally focus on single topics such as its effect on microbiota [10], crop yield [11] or economic assessments [12]. It should be noted that this study is unique in that multiple aspects are summarized while also elaborating on biochar decomposition rates and emission rates and how these play into benefits and optimization of biochar as a soil amendment for agricultural purposes. This study outlines how various types of biochar have benefits and play a role in agricultural soil amelioration, combating climate change and future endeavors of biochar use in agriculture (e.g., drought tolerance and addition to the circular economy model).

## 2. Historical Use of Biochar for Soil Amendment

The first evidence of biochar use as a soil enhancer was the Terra Preta, also known as the “Indian black earth”. Terra Preta is a type of soil initially discovered in Western Amazonia [1]. It can be recognized by its dark color, its high degree of aggregate stability provided by the presence of higher amounts of carbon and high nutrient content associated to an increased microbial presence [1].

The most important aspect of the Terra Preta is the fact that biochar was intentionally introduced to enrich the soil profile by prehistoric indigenous groups [1]. The use of biochar as a soil amendment has allowed the tropical rainforest to thrive and flourish [13]. The Terra Preta of the Amazon rainforest illustrates how poor soils can be amended to improve health and biomass fertility [2]. Tropical sandy soils are not naturally fertile, life is mostly supported by the forest canopy providing organic matter [2]. On the other hand, it has been found that the Terra Preta possesses higher levels of nutrients (nitrogen, potassium, calcium and phosphorus) as well as better soil structure and rigidity—a more secure organization of soil particles [2]. This improved structure is due to the highly stable organic carbon incorporated in this soil [2]. Lima et al. evaluated the various properties of the Terra Preta which led to the discovery of flakes from various types of mica in the soil sub-layers that were once used in pottery [13]. A clear deduction can be made whereby the clay ovens were indeed used to produce the char and that the clay particles entered in the soil along with the resultant biochar [13]. It is believed that this practice dated back to almost 2000 years [3]. The Terra Preta soils contain a vast array of microbial populations [4]. Particularly, there is a significant presence of acido-bacteria species, compared to other soils, the Terra Preta have 25% increased diversity of bacterial species [4]. This is so important because a variety of bacteria needs to be preset in soils to provide a nitrogen source to growing plants [4]. The discovery of the Terra Preta indicates that the original people of the Amazonia knew the technique of biochar production, or they probably intentionally introduced this material in the soil to improve fertility [13].

## 3. Biochar Production Process

**Figure 1:**
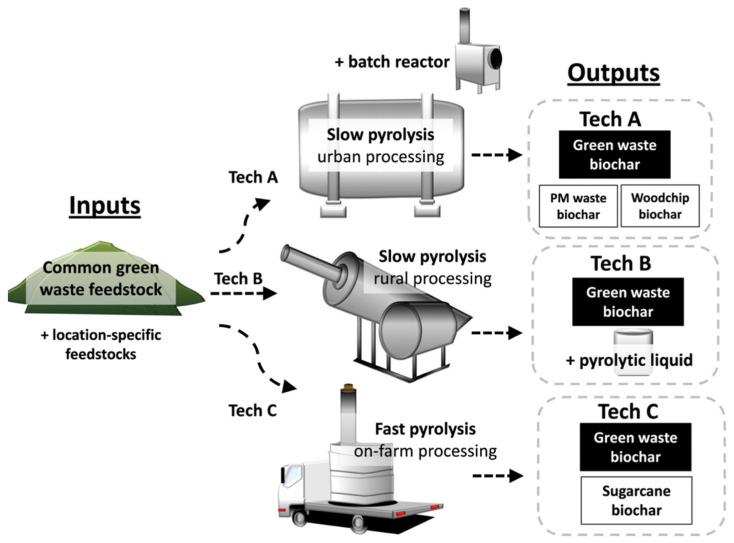
Simple pyrolysis reactor schematic [14]. [Copyright; PLOS ONE, 2016].

There are many ways to produce biochar and the method of production has a huge impact on the resultant characteristics. Pyrolysis is the thermochemical conversion of biomass in an oxygen starved/deprived atmosphere. Generally, pyrolysis process produces bio-oil, syngas and biochar [5]. In dedicated instruments, this process is carried out in the presence of an inert gas, typically nitrogen [6]. Figure 1 shows a simplified schematic diagram of a pyrolysis reactor [14]. This type of reactor can be used for either slow or fast pyrolysis [5]. The schematic portrays the input materials and output materials of the pyrolysis process.

The concepts of slow and fast pyrolysis refer to the heating rate of the process [7]. The heating rate determines if it is to be classified as fast or slow pyrolysis [8]. A few seconds to a few minutes indicate fast pyrolysis while minutes to hours indicates slow pyrolysis [6]. During pyrolysis, the natural polymers in biomass (cellulose, hemicellulose and lignin) undergo a few transformations [9]. These natural polymers will break apart, cross-link, and then fragment, each at different temperatures [8].

Slow pyrolysis produces more biochar and less bio-oil and syngas [15]. The opposite also tends to be true for the process of fast pyrolysis [15]. There is some evidence to suggest that specific surface area and pore volume are manipulated via these two processes, but it seems that other factors (such as maximum temperature and residence time) have a greater effect [16]. It’s important to note that biochar characteristics are not always homogenous even if the production method is similar [17]. The factors most affected by temperature are that of surface area and pH [18]. pH is affected by temperature since various functional groups, such as carboxyl, carbonyl and hydroxyl groups, are removed from biochar surface at different temperatures. While feedstock most strongly controls organic carbon content and mineral aspects [17], these effects seem to be true for biochar made from animal waste as well [19].

**Figure 2:**
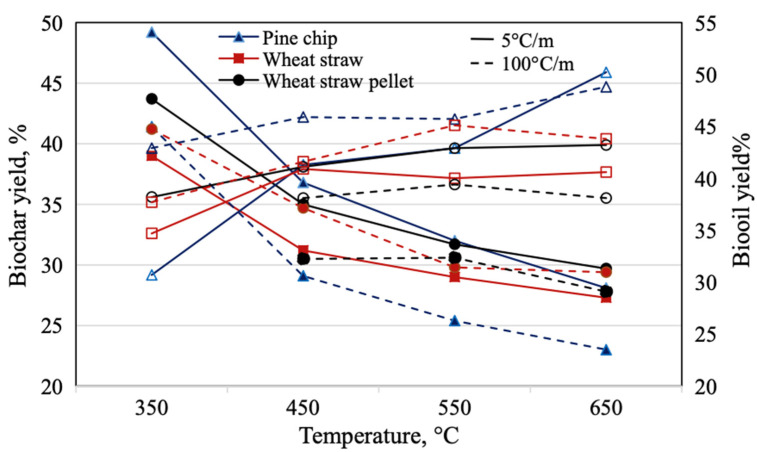
Effect of processing temperature on biochar and bio-oil yield [adapted with permission from ref. [15], [Copyright, Elsevier, 2017].

Low temperature biochar (below 550 °C) has a lower ash content and exhibits less crystalline structure which is more strongly affected by feedstock type [20]. As an example, Yoshida et al., 2008, compared biochar yields from two different feedstock sources; eucalyptus wood and banagrass (*Pennisetum pirpirem*). Under similar production conditions, mature banagrass produced higher biochar yields than that of eucalyptus wood [21]. It was postulated that the structure of banagrass is what formed its fixed carbon rate. The biochar yield can be manipulated through the feedstock selection process [21]. Typically, as temperature of pyrolysis increases, so does the available water holding capacity (WHC) [22]. Additionally, as seen in Figure 2, the biochar yield is dependent on both feedstock type and pyrolysis temperature [15]. The figure indicates how biochar yield is inversely related to bio-oil yield at varying temperatures. The general trend indicates that with an increase of pyrolysis temperature, biochar decreases while bio-oil yield increases. When looking at desirable biochar characteristics, higher degree of nutrients is a desired outcome [23]. Pyrolysis at temperatures above 400 °C burns off most of the nitrogen, potassium, and sulfur molecules and thus pyrolysis temperatures below allow those to exist in the resultant biochar [23].

Co-pyrolysis is the process of pyrolyzing more than one type of feedstock to create biochar and bio-oil [11]. One of these feedstocks could consist of a polymer such as polyethylene, mostly to improve quality bio-oil since a petroleum-based feedstock adds more characteristics typical of petroleum oil [11].

Hydrothermal carbonization (HTC), also referred to as wet-pyrolysis, is a process that creates a sub-category of biochar known as hydrochars, which can also be used for soil amendment [24]. Additionally, the authors indicate that it is necessary to determine the influence of hydrochars (and biochar in general) on plant growth since they are often used for soil amendment purposes to improve soil stability and pollutant removal [24]. The ability to predict biochar characteristics is important and it is possible when the process conditions are known [25].

Various characteristics of biochar are malleable when production conditions are altered [26]. Table 1 represents the properties of different biochar. The slow pyrolysis process produces the highest biochar yield, followed by hydrothermal carbonization and lastly co-pyrolysis producing the lowest biochar yields [27,28,29,30]. There seems to be no other discernable trends in N, O, H, S, P, K or pH. It is postulated that the differences are due to the feedstock type rather than the production conditions. The value of biochar in various applications depends on the biochar compositions.

## 4. Soil Applications of Biochar

Biochar addition can greatly improve soil integrity since soils require a certain degree of aggregates; solids and organic matter (or humus) to best provide a growing medium to plants [31]. Furthermore, a variety of particle sizes are required to maintain WHC along with a certain level of aeration [31]. Biochar can remediate the physical structure of poor soils. If soil is too compacted, then biochar incorporation may allow better aeration through its varying degree of porosity [31]. Biochar, when compared to fine, sandy soil types has a much larger surface area and higher degree of porosity [32]. There are also benefits to including the compost of biomass in combination with the biochar produced from that biomass [11]. The addition of compost and biochar proved to be just as favorable as biochar alone for plant yields due to the more rapidly degrading biomass providing a steady flow of nutrients for plant uptake until slow release of nutrients from the biochar began [11]. When soils are amended with biochar, there is a greater amount of oxidation-reduction reactions that take place within the soil matrix. The persistence of biochar in the environment and soils overtime is another benefit of biochar and can persist in fields over several years [23]. As such, biochar may not need to be re-applied yearly, thus proving to be a cost-efficient alternative.

Overtime soil organic matter content levels diminish. This occurs due to weathering, farming cultural practices and other anthropogenic activities [24]. The crystalline structure of biochar makes it particularly stable and withstands in soils [25]. Another interesting effect is that biochar’s addition to soils causes increased observation of ethylene, a major plant hormone used to regulate plant growth and ripening [26]. With an increase in ethylene being produced through biochar amendment, crop yields will increase [26].

### 4.1. Nutrients and pH

Biochar has the capability to retain and provide bioavailable nutrients for plant uptake. As an example, the potassium found within biochar is available for plant uptake [23]. Biochar can also have a variety of effects on soil pH, the degree to which is often dependent on the feedstock and production conditions [33]. There are several species of microbes (acidobacteria, nematodes and fungi such as mycorrhizae) found within soils [4]. Biochar has the capacity to help remediate the deficiencies seen in “problem soils”, they possess qualities such as poor aggregate stability, high salinity, extreme pH levels (too high or too low) or lacking in nutrients [34]. Problem soils can be defined as soils with poor properties (biological, physical, or chemical) that hinder plant growth [35]. The long-term health of soils can be improved with even a single application of biochar [35].

There are a variety of ways biochar can enhance soil health, and with this comes improved crop productivity [11]. One way this is accomplished is through improved microbial population diversity [10]. The refuge provided by biochar pores allows populations to propagate and it also fixes nitrogen for plant uptake [36]. This is particularly important for crops that are not able to fix their own nitrogen (in the case of non-legumes). It is particularly interesting to understand that the potassium found in biochar are already in forms that are available for plant uptake [23]. Biochar also makes nitrogen more available for plant uptake for crops that are not able to fix their own nitrogen [37]. Borchard et al., 2014 applied biochar at a rate of 15 g/kg of soil and saw an increase in maize yields, an increase in N, Ca and in maize leaves [38]. In addition, the carbon content in soil increased and temporarily decreased the soils pH level, although alkaline soils tend to be most favorable for common cash crops such as maize [38]. Another study by Agegnehu et al., 2016 also found soil properties to improve with the addition of biochar [11]. When analyzing the soil physicochemical properties, it was discovered that the treatments including biochar had increased nitrogen levels when compared to just fertilizer (1.16% vs. 0.15% soil nitrogen). With a biomass increase of 9–18%, it was found that there were higher levels of nitrogen within biomass leaves [11]. Using solid forms of carbon also allows soils to improve the level of nutrients present and how they are retained [39]. For soils that have undergone a high degree of weathering, nutrient retention is a particularly immense problem and they experience low Cation Exchange Capacity (CEC) due to the reduced mineral component found in that soil [39]. It also is related to improved WHC but the very surface bonding that occurs with improved CEC adds to the nutrient retention [39].

**Figure 3:**
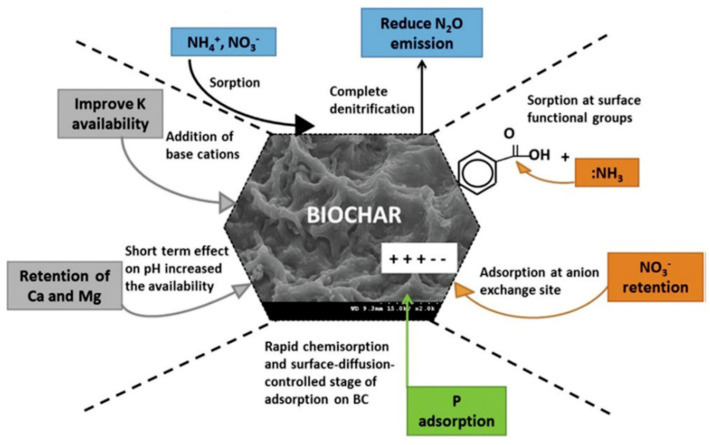
A variety of effects caused by biochar surface chemistry [33]. [Copyright; MDPI, 2020].

Another property of biochar is its ability to directly provide nutrients for plant uptake [2]. For example, the potassium that is present in biochar from its original feedstock is generally found in forms available for plant uptake [23]. As can be seen in Figure 3, whether pH increases or decreases after biochar amendment depends on the characteristics of the biochar [33]. Generally, biochars formed from agricultural residues tend to be more alkaline and therefore help increase the pH of soils [23]. These types of biochar have higher ash content which provides more basic salts to skew more alkaline [23]. In contrast, biochars created from animal residues, such as chicken litter or bovine manure, are significantly more acidic due to the functional groups they provide to biochar [34].

Extremely saline soils also pose a risk to crop production [40]. A field experiment spanning across 2 years discovered that a combination of wheat straw biochar and poultry manure was able to decrease the extreme salinity of central Chinese soils in order to improve the growth of maize [40]. What was most interesting here was the fact that leaf bioactivity increased, and the maize leaf sap had increased concentrations of nitrogen, phosphorus and potassium. The increased nitrogen coming from the chicken manure was taken up more easily by the biochar [40]. Soil salinity is reduced via the presence of Na^+^ ions, biochar binds to the Na^+^ ions and stops them from being taken up by the plant [41]. On acidic soils, corn stover and switchgrass biochar was found to increase soil pH and other properties over a period of 165 days, which is a relatively short amount of time [42]. The hydrogen and aluminum atoms free in soils cause acidity and limit crop growth [41]. They bind to essential plant nutrients and halt them from uptake [43]. Soils that have pH less than 5.0 are strongly acidic, which are the conditions where the most damage occurs [44]. The quality and yield of many crops are severely hindered at this point [44].

Even legumes, that fix their own nitrogen benefit nutritionally from the addition of biochar. The rate of nitrogen fixation of common beans increased after addition with biochar [45]. At a biochar addition of 90 g kg^−1^, the nitrogen fixation increased from 50% to 72% [45]. Even without the use of traditional chemical fertilizers, biochar can aid in the growth of crops [46]. In general, nitrogen retention increases with the addition of biochar. Biochar addition in a pot experiment on rice allowed the crop to take up more nitrogen fertilizer [47]. This resulted in 23–27% increase of nitrogen uptake and furthermore caused an 8–10% increase in rice yield. Interesting to note that without the application of nitrogen fertilizer, there was no positive yield effect on the rice [47].

The supplementation of biochar alone alters the bioavailability of various nutrient uptake [48]. One study was able to find that soil surface available phosphorous increased by 45% when possessing a viable amount of phosphorous [48]. Available nutrients are necessary to improve the yield of crops and thus increase food production [49]. This phenomenon is not limited to Zea mays, but increased levels of nutrients through biochar amendment leading to higher plant heights, higher wet biomass weight and higher dry biomass weight [49]. There are many cases in which the addition of biochar can reduce the motility of various fertilizer compounds [50]. Often this is in reference to less fertilization requirements, since less compounds of fertilizer are moving through and out of soils. For example, for the growth of sugar beet and potatoes, the optimal level of nitrogen fertilization was quite high at 300 kg ha^−1^ [51]. Referring to Table 1, the percent weight of nitrogen found in corncob biochar pyrolyzed slowly at 600 °C, was 4.25%. This would mean 7058.82 kg ha^−1^ of this corncob biochar could replace the traditional nitrogen fertilizer. Of course, this is a hefty amount of biochar but at the very least it means food producers could reduce the application rate of traditional fertilizers and replace a portion of it with biochar. It is more persistent in soils and will aid in reducing fertilizer motility as well. Compared to high temperature pyrolysis, low temperature pyrolyzed biochar tends to degrade at slower rates. This would mean an initial increase in biochar addition will last for multiple growing seasons instead of having to re-apply traditional fertilizers every growing period [15].

Due to the CEC of many biochars, surface functional groups, such as hydroxyl and carboxyl groups, can bind to nitrogen and phosphorous in many forms found in fertilizers [20]. For example, it was found that biochar formed from pine-chips at 300 °C created char with lower surface area (16.5 m^2^ g^−1^) along with lower cation exchange capacity (39.5 cmol kg^−1^ or centimoles per kilogram). This can account for the higher pH (8.2) since there were fewer functional groups on biochar surface to add to the acidity of the soil through amendment [20].

Greater nutrient retention by biochar is what increases the nutrient content of biochar amended soils [52]. It seems that biochar is effective at reducing the leaching of nitrogen, in the form of NH_3_. Wheat straw biochar applied at rates of 0.5% to 1% were enough to reduce N leaching in various forms. The following forms of nitrogen were assessed as a percentage, NH_4_^+^ by 11.6–24%, -N 13.2–29.7% and NO_3_^−^—N 14.6–26% [53]. This effect can be explained by the fact that biochar ends up covalently bonding to ammonia and helps retain it in soils [54]. Another study by Yao et al., 2012 discovered that peanut hull and pepperwood biochar pyrolyzed at 600 °C were able to sorb a variety of nutrients and reduce leaching in a column experiment [55]. Results showed that the amount of nitrate leaching reduced by 34%, ammonium by 34.7% and phosphate by 20.6% [55]. A study carried out on calcium rich soils known to have issue with nutrient retention indicated that bagasse biochar is effective at reducing nitrate leaching [56]. Pyrolyzed at 400–800 °C, this material was analyzed for its degree of CEC (measured at pH 7). In general, biochar application reduced the level of fertilizer needs [57]. With the addition of corn cob derived biochar and from fig tree derived biochar, the growth of maize was improved [12]. Produced via slow pyrolysis at 600 °C, these two types of biochar were lower in pH and their surface due to increased surface functionality. Through this greater surface functionality, there is a higher degree of free carboxylic carbon that will keep cations on biochar surface [12]. Using biochar can be an easy way to improve the organic matter content of soils [58]. When organic matter is not added, the microbiome will change to microbes that are predominantly anaerobic. This is a problem because this causes ammonium nitrogen to accumulate in the growing medium. This ammonium nitrogen when exposed to air quickly turns into a more mobile NO_3_-N. Overall, without the addition of biochar this can cause excessive leaching of various substances used as fertilizer [58].

### 4.2. Water Holding Capacity

One of the most important ways biochar is able to abate the effects of climate change induced drought is through its ability to retain water [59]. An effective example of this is use of biochar in sub-Saharan Africa to combat dry soils [60]. In this region, soils are generally poor because of higher concentrations of sand from granite-rock parent material [60]. Not only are these soils naturally dry, but also acidic in nature (often with a pH below 4) and produce low plant yields. The soils in this area are also stressed due to repetitive drought. The combination of these two effects produces massive food shortages to the area [60]. It is known that the WHC of biochar is most strongly influenced by the level of biochar porosity [11]. This porosity is made up of macro and micro pores [11]. This porosity is vital to increase soil water capacity, up to 14.6% increase of water content was found when biochar was used in combination with fertilizer [11]. Water is the primary vessel with which plants take up nutrients, additionally with lower WHC and improving soil health further increases other positive effects [39]. When there is a significant loss of water in soils, the plants residing will experience salt stress [61].

With the addition of biochar there is on average an increase of about 18% WHC [62]. It is known that biochar improves water-holding capacity through surface area and porosity [62]. Even across soil types, WHC improves with biochar amendment [63]. With just 9% addition of biochar (yellow pine wood pyrolyzed at 400 °C) there was a 100% increase of WHC [62]. Meaning that there was a doubling of WHC [62]. Another study discovered that the water holding capacity was increased by 30% when sunflower husk biochar was applied at 9.52% weight [64]. This is another example of how ~10% dry weight biochar application is generally optimal to remediate or improve soils [64].

### 4.3. Microbiome

Many factors of biochar affect microbial populations such as feedstock, pyrolysis conditions, particle size and soil properties [23]. There is evidence to suggest that biochar enrichment can help mycorrhizal and rhizobial populations at the root level [34]. With these microbial populations present, a variety of reactions take place within the soil matrix [20]. These occur at the interface between root hairs and microbes in soil. Through sorption, various organic compounds that are bonded to biochar structures can be used by the plant [23]. Addition of organic matter generally helps microbial species [65]. When assessing the health and abundance of mycorrhizal communities, biochar amendment has been shown to benefit them in the following ways; they provide a refuge through porosity, for species, they detoxify the soils of heavy metals and can alter the soil’s physic-chemical properties [66]. With the addition of biochar pyrolyzed at 350 °C, there were more bacteria (both gram negative and gram positive) in soils compared to the soil amended with biochars produced at lower or higher temperatures [36]. Additionally, greater aeration and more soil pores create the soil-water interface in which these microorganisms live [67]. Microbial populations can also aid in degradation of fertilizers thus, reduce issues of nutrient leaching [66]. Their presence in soils is vital for the health of food crops [65].

In general, the prognosis is good when it comes to improving the diversity and count of bacterial genes in biochar amended soils [68]. Chen et al., 2013 discovered that field of rice, biochar applied at 20,000 kg/ha and 40,000 kg/ha altered the microbiome populations. This shift included the favor of bacterial populations instead of fungal populations [68]. Another source indicated that there was no increase in microbial diversity but rather increased microbial biomass with the addition of biochar from various biomass sources in a meta-analysis by Li et al., 2020 [10]. It is thought that the reason for this is because bacterial groups in soils are more readily affected and sensitive to biochar, while fungi may not be [10].

## 5. Biochar Decomposition

Even though biochar in soil can serve as a carbon sink, biochar is not a permanent fixture because it degrades overtime [48]. For soil amendment application, biochar decomposition rates depend on the state of biochar, properties of soil and the climate [15]. When amended with biochar, soils were able to improve available phosphorous content minimally, although a very important finding, this only lasted for less than 6 months, and thus is a short-term negative effect [48]. Simulated aging of a field indicated that biochar produced from rice husk, modified with sulfur, was able to continually benefit soil health over 50 years of time. This was produced via slow pyrolysis at 550–600 °C [69].

**Figure 4:**
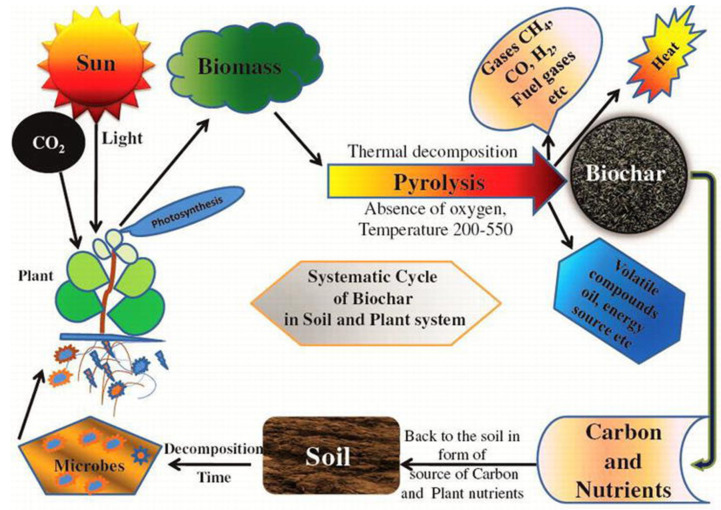
The cyclical and renewable nature of biochar as a soil amendment [73]. [Copyright; ItechOpen, 2020] Cycle of biochar decomposition and formation when amended into soils.

Pyrolysis time influences the resultant decomposition rate, as illustrated in Table 2 [70,71], even when temperature is kept the same. Biochar created via fast pyrolysis has a slower rate of decomposition in soil than biochar formed through slow pyrolysis [70,71]. It is important to then suggest fast pyrolyzed biochar for purposes of remediating problem soils in order to have the beneficial effects of amendment last longer. In this way, there is not only an environmental benefit but also an economic one. An example of biochar persistence in the environment is the aftermath of forest fires burning woody biomass that incorporates into the soil top layers [72]. Deadwood biomass from black spruce trees ignited through forest fires create a more stable form of solid carbon as opposed to raw biomass. Knowing that this raw carbonaceous matter is less stable than biochar ignited in the absence of oxygen indicates that one is able to harness the power of creating stable forms of solid carbon to remain in soils [72]. Since biomass is renewable, capturing it in a solid form allows soils to act as a carbon sink, when generally agricultural lands are not used in this way (Figure 4) [73].

## 6. Environmental Benefit

Since 1750, there has been an increase of 31% atmospheric CO_2_ [74]. Biochar has the ability to sequester this CO_2_ in the form of solid carbon in soils [75]. This allows it to use soils as a receptacle to sequester carbon. Carbon dioxide is not the only GHG that can be sequestered through biochar, nitrous oxide is also able to be sequestered [34]. This is because feedstock types that are higher in nitrogen content (including chicken litter, animal manure and municipal sewage sludge) pass that nitrogen content on to the plants [34]. It is important to understand the entire lifecycle of biochar to prove the sustainability of using this material. The carbon footprint of biochar must include production, persistence in soil, rate of degradation in soil and degree of soil fertility. An example of this was a study carried out in mainland China in which four paddy rice fields and 3 maize fields were amended with biochar [76]. The addition of biochar reduced the amount of released carbon by 18,479.35–37,457.66 kg of carbon dioxide [76], i.e., a reduction of 47% and 57% for both rice and maize, respectively [75]. Spokas and Reicosky, 2009 looked at 16 different types of biochar and their effect on GHG emissions in soils [77]. Feedstock, pyrolysis conditions and surface area all did not influence the amount of methane released from the growing medium [77]. In this study, after the addition of fertilizer, there would be a net increase of GHG emissions. Other studies prove that even when accounting for the GHG release during production, there remains a net decrease in GHG release when biochar is added to agricultural soils [77]. Over a 100-year period of this practice, the removal of the emissions from fallen branches reduced from 340 to 70 kg CO_2_ eq. MWh^−1^. This is a significant decrease in emissions from a very indirect and passive form of biomass breakdown [78].

Previously mentioned applications of biochar include filtration and truly this is a way in which to benefit the environment as well, to remove pollutants from the environment through porosity and CEC of the material [79]. Toxin removal is accomplished through waste-water treatment, reduced GHG emissions, controlled degradation of land (through adding stability and aggregates to soils), reduced nutrient leaching, release controlled fertilizer and heavy metal and pollutant removal [79]. Previously mentioned Ebeheakey et al., 2018 noted a great reduction in lead acetate, ferric chloride, saponins, flavonoids and triterpernoids after biochar amendment [80]. At 12 weeks post amendment, the soils did not contain any of these substances [80]. Additionally, it is possible that altered biochar can more effectively sorb pollutants from waterways. The high porosity of slow pyrolysis biochar formed from cotton and sewage sludge had maximum sorption ability of 1.761 mg g^−1^ and 2.586 mg g^−1^ [81]. Another source indicated that bamboo biochar was able to reduce nitrogen species from water [82]. The maximum sorption ability in this case was 10.35 mg g^−1^ in the unaltered biochar but greater for modified biochar [82].

Global warming potential (GWP) indicates how much energy the emissions of one ton of carbon dioxide absorbs [24]. As can be seen in Table 3, the miscanthus feedstock produced the lowest GWP while aiding in using soils as a carbon sink [15]. Meanwhile, peat moss feedstock has the most GWP. Observing the intermediate GWP value for the co-pyrolyzed biochar (formed from both miscanthus and peat moss feedstock) is proof of the predictability of biochar qualities based on feedstock, production type and conditions. The amount of stored carbon increased while decomposition rate decreases. Obstinately, a lower amount of carbon can be stored with a greater decomposition rate [15]. The effects of biochar on crop yields is also a fundamental benefit that overall aids in protecting the environmental system [42].

As seen in Figure 4, biochar can not only reduce CO_2_ emissions but also reduce nitrous oxide [33,72]. If food producers can increase crop yields within current crop land area and without expanding agricultural lands, this helps protect current forested land [42]. Carbon content in soils depleting is already a concern in agroforest ecosystems [83]. For example, in India, rain fed crops in fact rely upon soil organic matter in the form of carbon to thrive. For every addition of 1 Mg ha^−1^ of soil organic carbon (SOC) there are grain crop increases. 1000 kg ha^−1^ of SOC increases the yield of groundnut by 13 kg ha^−1^, finger millet by 101 kg ha^−1^, sorghum by 90 kg ha^−1^, pearl millet by 145 kg ha^−1^, soybean by 18 kg ha^−1^, and rice by 160 kg ha^−1^ [83]. This relatively small addition of organic matter in the form of carbon allows for not only crop yield increase but also reduction of GHG emissions. Even in instances where biochar addition does not decrease GHG emissions, there still exists an increase in crop yields without any increase in those same emissions [84]. When straw derived biochar was amended into sandy-loam soils (low in organic matter) a five-year wheat and maize crop rotation resulted in a decrease of N_2_O emissions, crop yields did increase but overall GWP did not decrease [84]. This is due to biochar production emissions essentially. Despite this, the evidence still remains that the outcome of amendment is either neutral or beneficial in terms of crop yield, GWP, emissions rates and SOC rates. Other aspects of conservation farming exist, these include no tillage, crop rotation, permaculture, etc. [85]. The issue is that none of these strategies alone have been able to offset emission rates and SOC degradation rates in any meaningful capacity. Therefore, it should be highlighted that biochar has yet to be used in a widespread way in order to accomplish the same goals [85]. Furthermore, when discussing aspects of traditional agriculture, there exists many constraints when utilizing typical chemical fertilizers. Yes, the addition of biochar in soils reduces the immediate GWP as compared to compost, but when looking at even broader analysis, overall GWP is also reduced [86]. Therefore, it can be said that optimization of fertilizer application is important to control GHG emissions [87].

## 7. Economic Benefit

Even though biochar seems costly, its agricultural applications provides long-term economic benefits. An economic assessment carried out by Keske et al., 2019 was able to determine that biochar application for agricultural purposes, had a 99% probability of becoming profitable [12]. When using biochar created from forest biomass waste (in this case black spruce) to grow beets, crop yields increased [12]. Biochar was applied at 10,000 kg/ha, and the resultant beet yields increased from 2900 kg/ha to 11,004 kg ha^−1^ [12]. The net return for this process was up to $4953 ha^−1^. Conversely, the same study indicated that the costs of application were covered for beet production but not potato production [12]. In general, when being produced from waste biomass, biochar production is economically beneficial [22]. It is also important to note that using soils as a carbon sink creates a more economically sustainable method to manage waste [88]. Poland is an example of a nation that would benefit greatly from establishing biochar production within a circular economy model [89]. Additionally, some work has been carried out to determine that biochar use in soils can act as carbon sequestration in the Polish climate and soil type which is very promising [89].

As mentioned in Table 3, the use of different production conditions can help create a type of biochar that is more persistent in soils and thusly will not require yearly application allowing for more fiscal saving. However, the current market price of biochar may restrict its use for either amending soil or for energy generation and this requires exploration.

## 8. Discussion

Despite all the benefits biochar can provide, there is controversy surrounding biochar amendment in agricultural soils. To keep up with growing food demands, it is necessary to have a focus on improving the yield of staple crops. Additionally, Chan et al., 2007 carried out a pot trial to study green waste biochar effects on radish yields (Raphanus sativus) [88]. The biochar was applied at 10, 50 and 100 t/ha. The soils the study was carried out on had a history of regular cropping and were of the soil type alfisol. In this case the yield of radish was not improved, even at the highest application rate of 100 t/ha. The most interesting interaction involved nitrogen. When biochar was applied together with nitrogen fertilizer, the yields improved [5]. Time of residence for biochar can also vary. As an example, some sources indicate that wood biochar can have a longevity of anywhere from 100 to 1000 years [45]. This broad range means that determining re-application rates for croppers can be difficult. There is very little data concerning the time of residence for other types of biochar as well [45]. Jeffery et al., 2015 included a study in which the application of biochar was not able to improve hydrological qualities of a sandy soil [90]. Critique of this study may include the fact that the study site in question was in the Netherlands where soil quality is often thought to be of the highest caliber. These kinds of confounding results are a major constraint when it comes to biochar soil amendment. El-Nagger, 2019 analyzed multiple sources in order to create a review of the outcome of biochar amendment on soils with low fertility [91]. The benefits of biochar amendment depend on the product used for the specific situation. Particular situations require specialized biochar products, whether it is activated or not [21].

**Figure 5:**
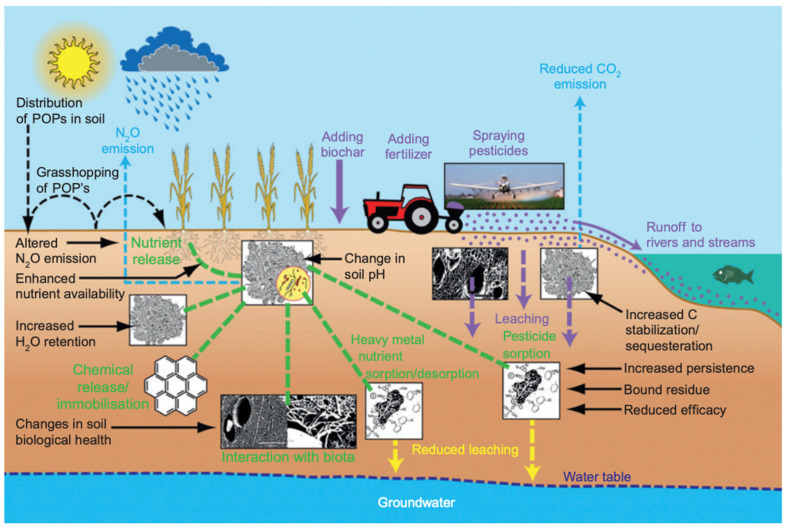
The multitude of benefits biochar soil amendment provides. [adapted with permission from ref. [92], [Copyright, Elsevier, 2011].

Much of the soils found on earth are highly acidic and would benefit from the addition of biochar [92]. This is as a result of mainly anthropogenic activities [93]. Biochar tends to be more alkaline and helps increase the pH in order to reduce acidity [94]. When fava beans and turnip rape were grown with the addition of pine tree biochar, there was an increase of yield as a result of increased water holding capacity [93]. When categorizing soil types, 38% of tropical regions were able to benefit from biochar amendment while only 10% of temperate soils saw the same fertility benefits. However, there is very little work carried out to fully understand why these differences occur. N_2_O emission could be triggered due to increased nitrogen fixation by microbes, whose populations are stimulated by biochar addition [92]. A lot of the benefits of biochar addition into soils are illustrated in Figure 5. For example, using soils as a carbon sink to reduce GHG emissions, reduced fertilizer leaching into waters and soils, or improved nutrient availability for crops, just to point out a few [92]. Future endeavors of biochar use in agriculture include climate change mitigation, drought tolerance and addition to the circular economy. As discussed earlier, the method of capturing carbon in a solid form helps reduce, or at the very least, delay GHG emissions. If nations that participate in carbon tax programs (such as Canada and Zambia) make biochar readily available to food producers, this system could continue to thrive [95]. While carbon taxes or carbon credits are seemingly focused on manufacturing processes, agriculture remains as one of the most emission-heavy industries [96]. This is an unexplored option to reduce emissions without penalizing other emission-heavy industries. While certain businesses may leave nations in which penalization may occur, food production will remain sturdier in their locations due to the inherent need for food. No matter the economic situation, food production is vital, and agriculture remains.

**Figure 6:**
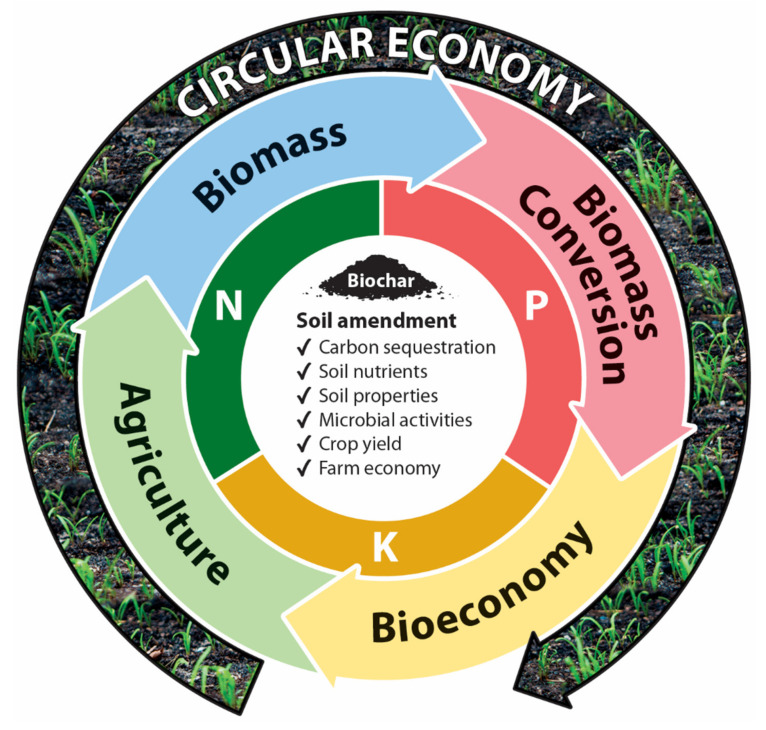
The role biochar plays in the circular economy model.

Furthermore, biochar very effectively fits into the circular economy model. The biochar production process can play a role in multiple industries, but biochar production most strongly has a role in agriculture. The growth of food will produce agricultural residue, which when valorized/polymerized forms biochar as one of the co-products. After soil amendment, there is an improvement of soil properties, nutrient retention, microbial activity and crop yield as demonstrated in Figure 6. Overall, this benefits the farming economy. In addition, reduction of waste from the agricultural sector will occur. In fact, a case study surrounding a small-scale olive farm enacted what was essentially a circular economy model regarding olive farming residues and bioenergy production [97]. This is an example of biochar providing value-added products into the agricultural economy to stimulate the circular economy model.

## 9. Conclusions

Biochar can improve agricultural soils in a variety of ways. These methods include, but are not limited to, improving water holding capacity, improving soil stability through addition of aggregates and solids, increasing microbiome populations and controlling fungi populations, reducing the need for fertilizer and reduce fertilizer leaching. One is also able to acknowledge the immense benefits to crop yields, the reduced GHG emissions and the role played in the circular economy model. Aside from agriculture, biochar can be used for water filtration purposes, removing heavy metals from the environment, and removing pharmaceuticals from the environment. It is essential for the future of biochar application to learn how to manipulate biochar properties so as to tailor the amendment to each region, climate, crop type and soil. Overall, the literature indicates that biochar has beneficial effects on soil quality and crop yields, but possible constraints need to be explored. The variability of biochar properties should be viewed as its best asset. Biochar seems to be a potential material that can be tailor-made to solve unique agricultural challenges.

## Figures and Tables

**Table 1:** Properties of different biochar.

**Feedstock**	**Production Conditions**		**Component, %**						**Reference**
		**C**	**N**	**O**	**H**	**S**	**P**	**K**	
Rice-straw	Fast pyrolysis 800 °C	36.2		39.8					[27]
Corn cob	Slow pyrolysis 600 °C	79.1	4.25					10.1	[28]
Corn stover	Slow pyrolysis 600 °C	69.8	1.01			0.181	2.461	9.95	[28]
Peanut hull	Slow pyrolysis 400 °C	65.5	2.0			0.00162	0.0015	10.0	[28]
Corn stover	Slow pyrolysis 300 °C	59.5	1.16			0.137	1.705	7.33	[28]
Corn residue (Stover and cob)	HTC 260 °C (30 min)	57.51 ± 1.11	1.62 ± 0.04	35.12 ± 1.09	0.23 ± 0.02				[29]
Rice husk + high density polyethylene	Co-pyrolysis 300 °C	46.802 ± 0.960	0.670 ± 0.003		0.036 ± 0.002				[30]

**Table 2:** Decomposition rates of biochar from eucalyptus and oak feedstock produced under varying conditions.

Feedstock	Production	Decomposition Rate
Eucalyptus	Pyrolyzed 450 °C, 0.7 h	0.0039 [70]
Eucalyptus	Pyrolyzed 450 °C, 3 h	0.0081 [70]
Oak	Pyrolyzed 450 °C, 3 h	0.003 [71]
Oak	Pyrolyzed 450 °C, 0.7 h	0.0047 [71]
Eucalyptus	Pyrolyzed 450 °C, 0.7 h	0.0049 [70]
Eucalyptus	Pyrolyzed 450 °C, 0.7 h	0.0039 [70]

**Table 3:** Global warming potential (GWP) of different processed biomass applied for soil amendment [15].

Feedstock	Production Method	Application	GWP/t Feedstock (Kg CO_2_ eq)
Peat moss and Miscanthus	Hydrothermal carbonization	Soil amendment	79.51
Miscanthus	Hydrothermal carbonization	Soil amendment	−320.86
Peat moss	Hydrothermal carbonization	Soil amendment	714.64

## Data Availability

The data presented in this article is available on request from the corresponding author.
